# Glutathione in the Brain

**DOI:** 10.3390/ijms22095010

**Published:** 2021-05-09

**Authors:** Koji Aoyama

**Affiliations:** Department of Pharmacology, Teikyo University School of Medicine, 2-11-1 Kaga, Itabashi, Tokyo 173-8605, Japan; kaoyama@med.teikyo-u.ac.jp; Tel.: +81-3-3964-3793

**Keywords:** glutathione, cysteine, excitatory amino acid carrier 1, glutamate transporter-associated protein 3-18, miR-96-5p, neurodegeneration

## Abstract

Glutathione (GSH) is the most abundant non-protein thiol, and plays crucial roles in the antioxidant defense system and the maintenance of redox homeostasis in neurons. GSH depletion in the brain is a common finding in patients with neurodegenerative diseases, such as Alzheimer’s disease and Parkinson’s disease, and can cause neurodegeneration prior to disease onset. Excitatory amino acid carrier 1 (EAAC1), a sodium-dependent glutamate/cysteine transporter that is selectively present in neurons, plays a central role in the regulation of neuronal GSH production. The expression of EAAC1 is posttranslationally controlled by the glutamate transporter-associated protein 3–18 (GTRAP3-18) or miR-96-5p in neurons. The regulatory mechanism of neuronal GSH production mediated by EAAC1 may be a new target in therapeutic strategies for these neurodegenerative diseases. This review describes the regulatory mechanism of neuronal GSH production and its potential therapeutic application in the treatment of neurodegenerative diseases.

## 1. Introduction

In 1888, de Rey-Pailhade described a substance with the property of reducing sulfur to hydrogen sulfide in extracts from yeast; he named this molecule ‘*philothion*’ [1], meaning “love of sulfur” in Greek [2,3]. Heffter suggested that cysteine (Cys) was involved in this molecule [4], although the structure of this substance was not revealed until the 1920s. Since that time, many researchers have been engaged in the extraction and synthesis of glutathione (GSH), including three Nobel laureates. In 1921, Frederick G. Hopkins isolated a dipeptide containing glutamate (Glu) and Cys from yeast and animal tissues, and named it ’glutathione’ [5]. However, de Rey-Pailhade thought that glutathione was a side chain of philothion, which was speculated to be a protein [3]. Other researchers isolated GSH from yeast, blood, and liver, and suggested that GSH was not a simple dipeptide composed of Glu and Cys [6]. Ultimately, in 1929, Hopkins redetermined that GSH was a tripeptide containing Glu, Cys, and glycine (Gly) [7]. In the same year, Hopkins was awarded the Nobel Prize in Physiology or Medicine for his work on vitamins, not GSH. Also in 1929, Edward C. Kendall crystallized GSH and identified its chemical structure [8]; Kendall went on to be awarded the Nobel Prize in Physiology or Medicine for his work on corticosteroids in 1950. Finally, Vincent du Vigneaud first reported the synthesis of GSH in 1936 [9]; he was awarded the Nobel Prize in Chemistry in 1955 for his work on biochemically important sulfur compounds, especially for the first synthesis of oxytocin. As evident from this thumbnail history, GSH inspired the interest of some of the most prominent researchers of the early twentieth century. More than 130 years after its discovery, GSH is still a promising therapeutic target for the treatment of neurodegenerative diseases. In this review, I will discuss the functions and regulatory mechanisms of GSH, with a special focus on its neuroprotective role against oxidative stress in the brain.

## 2. GSH Function

GSH is a major antioxidant that maintains the homeostasis of redox states in cells, and plays important roles in maintaining the physiological functions of all cells in vivo. The thiol (sulfhydryl, SH) residues play an important role in maintaining the redox state homeostasis intracellularly. In mammals, Cys and methionine (Met) are particularly important as thiol-containing amino acids [10], but GSH is the most abundant thiol-containing substance (derived from a non-protein) in all kinds of cells. The functions of GSH in living cells are diverse, and include roles in maintenance of the intracellular antioxidant system, redox balance, Cys transport/storage, cell signaling, regulation of some enzyme activities, gene expressions, and cell differentiation/proliferation [11]. GSH is especially abundant in the liver and kidney [12], both of which utilize the transsulfuration pathway to produce Cys from Met via homocysteine [13], while it is present at lower levels in the brain, where the regulatory system for GSH synthesis is independent of that in peripheral tissues. Therefore, the molecular mechanisms underlying GSH dysfunction in the brain differ from those in peripheral tissues.

**Figure 1:**
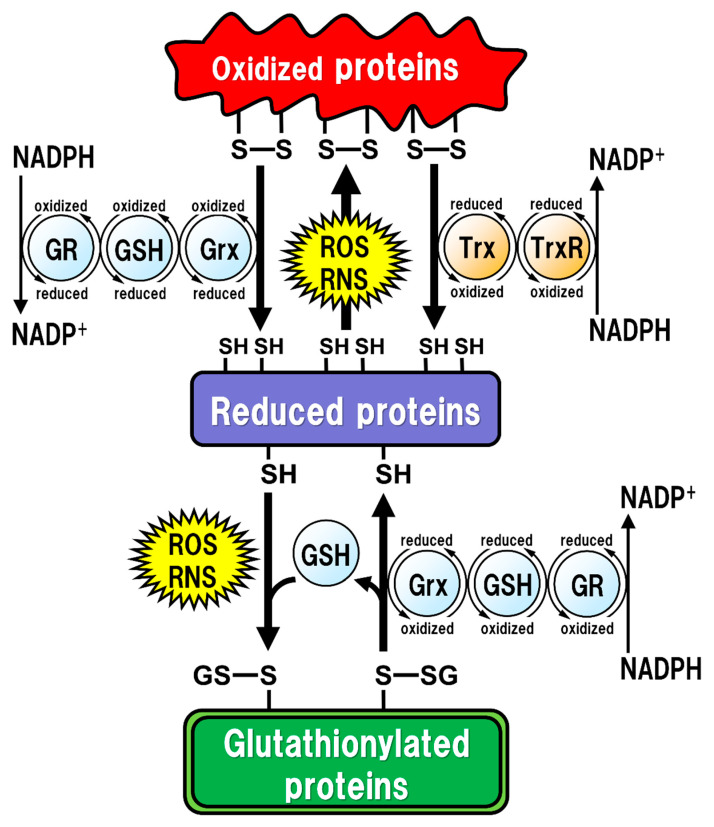
Regulation of the intracellular protein redox state by glutathione (GSH), glutaredoxin (Grx), and thioredoxin (Trx). Reactive oxygen species (ROS) and reactive nitrogen species (RNS) cause protein dysfunction, which is induced by the oxidation of thiol (SH) residues to form disulfide (S-S) bonds in the active site. Grx and Trx regulate protein function by reducing the S-S bonds of the substrate proteins. Consequently, Grx and Trx themselves result in the oxidized forms, which are reduced back by GSH and Trx reductase (TrxR), respectively. Oxidized GSH (GSSG) is reduced back to GSH by GSH reductase (GR). Both oxidized TrxR and GR are reduced by receiving electrons from nicotinamide adenine dinucleotide phosphate (NADPH). Under oxidative stress conditions, GSH can bind to cysteine residues (GS-S) in a process known as ‘*S*-glutathionylation’ to prevent the irreversible dysfunction of the proteins. Grx also functions in the deglutathionylation of the GS-S containing proteins to resume protein functions under physiological conditions.

All of the major biological processes of GSH involve the redox state of the thiol residue within the GSH molecule. Two molecules of GSH are oxidized to produce one molecule of GSH disulfide (GSSG) in order to eliminate reactive oxygen species (ROS)/reactive nitrogen species (RNS), or to maintain intracellular redox homeostasis, and GSSG can be reduced back to two GSH molecules via reaction with GSH reductase (GR). The intracellular GSH/GSSG ratio is 100 or more in the steady state, but decreases to 10 or less under oxidative stress conditions [14]. Proteins oxidized by ROS/RNS are reduced by glutaredoxins (Grxs) or thioredoxins (Trxs) [15] (Figure 1). Functionally, Grxs and Trxs share many common features; however, Grxs are more versatile than Trxs in terms of their substrate selectivity and reaction mechanisms [16]. The isoforms of Grxs and Trxs are known as cytosolic Grx1 and Trx1, and as mitochondrial Grx2 and Trx2 in mammals [16,17]. These isoforms are involved in controlling intracellular redox signaling for the cellular processes of apoptosis and proliferation [17,18]. Grx2 and Trx1 are also found in the nucleus. Many transcription factors are known to undergo reduction by Grxs and Trxs. In order for a transcription factor to bind to DNA, the thiol groups of Cys residues in the DNA-binding site should be in the reduced form. The thiol groups mainly exist in their oxidized forms, and are reduced in the process of becoming activated to enable DNA binding. Subsequently, the oxidized Grxs and Trxs are reduced back via reaction with GSH and Trx reductase (TrxR), respectively. GSH functions as an enzyme cofactor for Grxs, which are low-molecular-weight redox enzymes that are also known as thiol transferase, to maintain cellular redox homeostasis, and also acts as the primary reductant of the disulfide bonds of oxidized proteins [15] (Figure 1). However, excessive oxidative stress causes irreversible oxidation of the thiol residues and impairs cellular protein function [19,20]. In particular, Cys residues in active sites or functional motifs of intracellular proteins are important for their protein functions. Oxidative stress by ROS/RNS on the Cys residues in proteins can cause irreversible modifications that lead to critical dysfunction of the proteins [21]. GSH can also react with intracellular protein thiol residues to protect protein functions related to enzyme activities, DNA binding by transcription factors, and protein stability [22,23,24]. Therefore, under such oxidative stress conditions, GSH reversibly binds to the thiol residues to prevent irreversible changes in proteins due to oxidative stress. This post-translational modification, called “glutathionylation”, is reversible and protects the intracellular signal transduction system against oxidative stress [19] (Figure 1). The caspase family of Cys proteases, which induces cell apoptosis, would be a potential target for glutathionylation. Caspase-3, an important regulator of apoptotic responses, can undergo glutathionylation, leading to the inactivation of caspase-3 by GSSG in a dose- and time-dependent manner [25], suggesting that apoptosis can be regulated by glutathionylation. Once the cellular environment is free from oxidative stress, the disulfide bonds in the proteins are reduced back by Grx to function normally under physiological conditions [15]. Thus, the regulation of the redox state by intracellular GSH is extremely important for maintaining cellular functions under both physiological and pathological conditions. Especially in the brain, the regulatory mechanism of GSH function is more fragile in neurons than in glial cells, and intracellular GSH levels are also lower in neurons than in glial cells [26]. Under some pathological conditions, decreased GSH levels could have critical influences on neuronal activities, leading to neurodegeneration.

## 3. Oxidative Stress in the Brain

GSH occupies approximately 95% of non-protein thiol groups in vivo and is ubiquitously present in mammalian cells at concentrations of 0.5 to 10 mM, depending on the tissues [27,28]; these concentrations are 10 to 100 times higher than the concentrations of Cys in mammalian cells [29]. GSH is the major intracellular thiol compound, and is made from Glu, Cys, and Gly by two-step enzymatic reactions requiring ATP. The first step of these reactions is mediated by Glu-Cys ligase (GCL), and the second by GSH synthetase (GSS). In intracellular GSH synthesis, GCL can be the rate-limiting enzyme under the condition that all substrates are sufficiently present for the reactions, but the intracellular Cys concentration is much lower than those of Glu or Gly under physiological conditions, suggesting that the Cys availability is limiting for GSH synthesis [30,31]. GCL is comprised of both a catalytic (GCLc) and a modulatory (GCLm) subunit. GCLc is responsible for all of the enzyme activity of GCL, which is regulated via feedback inhibition by GSH [32]. Most GSH is present in the cytoplasm, where it is synthesized in mammalian cells [33]. Although mitochondria contain about 5–15% of all the GSH in the cell [34], they cannot synthesize GSH by themselves because they lack GCL [33]. The finding that GCLc- or GSS-deficient mice are non-viable in the embryonic period [35,36], while GCLm-deficient mice are viable and fertile with decreased GSH levels in the tissues compared to those of wild-type mice [37], suggest that GSH is essential for embryogenesis. 

The highest GSH concentration in the body is in the liver (about 5 to 10 mM) [12], but hepatocytes can also produce Cys for GSH synthesis from Met via the transsulfuration pathway [38]. GSH in the liver is then released systemically, but it is decomposed in the blood, with the result that the blood GSH concentrations (approximately 2 to 20 µM) are hundreds to thousands of times lower than those in the liver [28]. In addition, GSH cannot directly enter the brain due to the existence of the blood-brain barrier (BBB). Moreover, extracellular GSH cannot be directly transported into the cells, and thus the three amino acids used as substrates for GSH synthesis should be taken up into the cells via transporters. 

**Figure 2:**
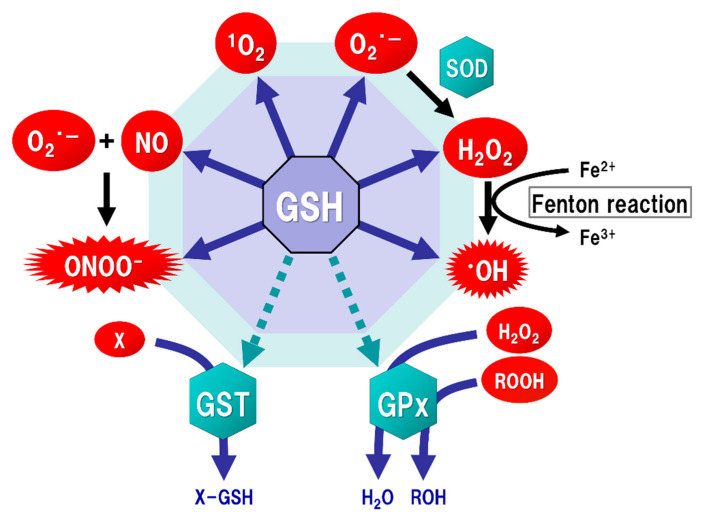
Function of glutathione (GSH) as an antioxidant. Mitochondria generate superoxide (O_2_·^−^), which reacts with nitric oxide (NO) to form peroxynitrite (ONOOˉ), a typical reactive nitrogen species (RNS) that is a potent inducer of cell death. O_2_·^−^ is catalyzed to hydrogen peroxide (H_2_O_2_) by the reaction of superoxide with superoxide dismutase (SOD). H_2_O_2_ reacts with Fe^2+^ (Fenton reaction) to form a highly oxidizing radical, hydroxyl radical (·OH). GSH can directly act as an antioxidant (solid arrows) by non-enzymatically reacting with NO, singlet oxygen (^1^O_2_), O_2_·^−^, H_2_O_2_, ·OH, and ONOOˉ. GSH can also indirectly serve as an enzyme cofactor for detoxification (dotted arrows). H_2_O_2_ is catalyzed to water and oxygen by GSH peroxidase (GPx), which requires GSH as an electron donor to react with H_2_O_2_ and hydroperoxides (ROOH). GSH-S-transferase (GST) can detoxify various xenobiotics (X) via GSH conjugation to excrete toxic compounds from the cell.

The brain tissue is generally rich in unsaturated fatty acids, which are targets of oxidative stress, and has relatively low levels of antioxidants or antioxidant enzymes. ROS, such as singlet oxygen (^1^O_2_), superoxide (O_2_·^−^), and hydroxyl radicals (·OH), are endogenously produced by mitochondria, cytochrome P450 metabolism, peroxisomes, and inflammatory cell activation. Mitochondria generate most ROS, including O_2_·^−^, into the matrix and the intermembrane space via the electron transport chain. The steady-state concentration of O_2_·^−^ is about 5–10 times higher in the mitochondrial matrix than in the cytoplasm or nucleus [39], but the mitochondrial matrix contains Mn-superoxide dismutase (SOD), which can react with O_2_·^−^ to form hydrogen peroxide (H_2_O_2_) (Figure 2). In addition, O_2_·^−^ leaked into the cytoplasm reacts with Cu/Zn-SOD (SOD1) to form H_2_O_2_. H_2_O_2_ is toxic to eukaryotic cells at concentrations of 0.1 to 1 × 10⁻^3^ M, but the reaction with catalase or GSH peroxidase (GPx) can decompose H_2_O_2_ to oxygen and water. As a result, the concentrations of H_2_O_2_ in mitochondria are maintained in the range of 10⁻⁹ to 10⁻⁸ M [15]. Such high concentrations of H_2_O_2_ are unlikely to occur under physiological conditions in vivo. However, overproductions of both O_2_·^−^ and H_2_O_2_ can be induced by mitochondrial dysfunction [40]. The increased H_2_O_2_ produces ·OH, which possesses the highest reactivity and the strongest oxidizing power among ROS, via the Fenton reaction (Figure 2). In addition, O_2_·^−^ reacts with nitric oxide (NO) to generate peroxynitrite (ONOOˉ) (Figure 2), which targets DNA, proteins, and lipids, causing DNA damage, dysfunction of enzymes, receptors, transporters, and membrane channels, as well as protein aggregation, mitochondrial dysfunction, and lipid peroxidation [15]. ONOOˉ is produced approximately one million times faster, and can spread approximately 10,000 times farther over cells, than ·OH [41]. ONOOˉ is more globally toxic within tissues than ·OH, whose toxicities are limited to the local area inside the cells [42]. GSH acts protectively against oxidative stress by reacting directly with NO, O_2_·^−^, H_2_O_2_, ·OH, and ONOOˉ (Figure 2). GSH also acts as an enzyme cofactor for GPx to degrade H_2_O_2_ and hydroperoxides (ROOH), and is involved in detoxifying electrophilic xenobiotics via GSH-*S*-transferase (GST) [43] (Figure 2). From these protective functions, GSH is considered to play an important role not only under physiological conditions but also under pathological conditions induced by oxidative stress in order to maintain the homeostasis of cell functions.

## 4. GSH Synthesis in Neurons

In in vitro studies, GSH levels in neurons are lower than those in astrocytes [26], and are increased when the neurons are co-incubated with astrocytes [44]. Neuronal GSH synthesis is supported by astrocytes, which supply GSH precursors to neurons. Notably, neuronal GSH levels in vitro are increased by the administration of Cys, but not Glu, Gly, or cystine, the latter of which is formed by two Cys molecules with a disulfide linkage [44,45]. Both Cys and Met are major sources of mammalian thiols [10], and Cys is an important substrate for GSH synthesis in neurons [46], while astrocytes can utilize both Cys and cystine for their GSH synthesis [45]. The activity of GCL, the rate-limiting enzyme for GSH synthesis, was upregulated in neurons co-cultured with GSH-depleted astrocytes, but the neuronal GSH levels were not increased [47]. These findings suggest that not only neuronal GCL activity, but also the astroglial supply system with Cys-containing precursors, is important in maintaining neuronal GSH levels.

**Figure 3:**
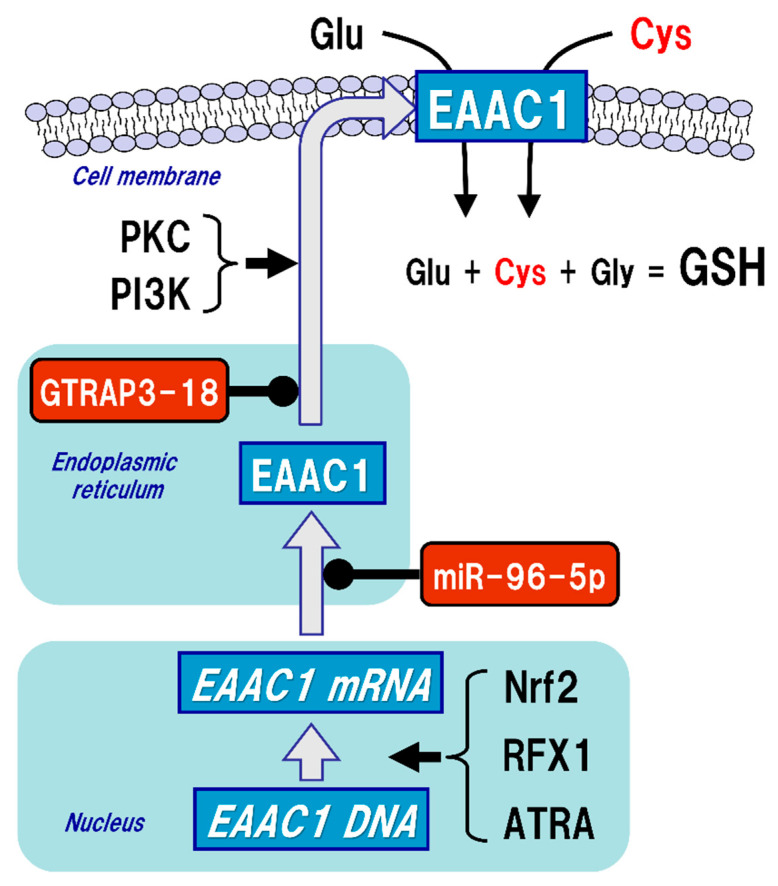
Regulation of excitatory amino acid carrier 1 (EAAC1) expression. Glutathione (GSH) is a tripeptide synthesized from glutamate (Glu), cysteine (Cys), and glycine (Gly). Neuronal GSH synthesis relies on intracellular Cys but not Glu or Gly level. Cys uptake (red font) is subjected to the regulation of both gene expression and post-translational modifications of EAAC1 under facilitative (arrow) and suppressive (black circles) controls. EAAC1 gene expressions are promoted by nuclear factor erythroid 2-related factor 2 (Nrf2), regulatory factor X1 (RFX1), and all-trans-retinoic acid (ATRA). Protein kinase C (PKC) and phosphoinositide 3-kinase (PI3K) activations increase the EAAC1 expression on the plasma membrane. Glu transporter-associated protein 3-18 (GTRAP3-18) and miR-96-5p post-translationally suppress the protein expression of EAAC1, leading to decreased Cys uptake and subsequently decreased GSH synthesis in neurons.

The uptake of Cys into neurons is mainly mediated by excitatory amino acid carrier 1 (EAAC1, in rodents), also known as excitatory amino acid transporter type 3 (EAAT3, in humans) (Figure 3). Five types of EAAT have been reported so far, and their expressions differ depending on the cell type. In the brain, GLAST (also known as EAAT1) and GLT-1 (also known as EAAT2) are primarily distributed in astrocytes, whereas EAAC1 is exclusively expressed in neurons. EAAT4 and EAAT5 are distributed in cerebellar Purkinje cells and neurons of the retina, respectively [48]. All of these transporters can take up extracellular Glu into the cells, but unlike GLAST and GLT-1, EAAC1 can also transport Cys with the same efficiency as Glu [49]. Based on the experimental results using a mutation model of EAAC1, it has been considered that the mechanisms of Glu and Cys uptake by EAAC1 are independent of each other [50]. There were no significant changes in extracellular Glu concentrations in an EAAC1-knockdown animal model [51]. GLAST and GLT-1 act as Glu transporters in glial cells in vivo and are involved in the regulation of Glu concentration in synaptic clefts, whereas EAAC1 is not involved in the regulation of extracellular Glu levels in synaptic clefts, but rather in the regulation of GSH production via extracellular Cys uptake. Moreover, EAAC1-deficient mice exhibit decreased brain GSH levels, vulnerability to oxidative stress in the hippocampus, and age-related learning dysfunction [52]. EAAC1-deficient mice also showed age-dependent loss of dopaminergic neurons in the substantia nigra pars compacta accompanied by increased oxidative stress [53]. EAAC1 is responsible for approximately 70–80% of Cys uptake in neurons [54], and can transport 10- to 20-fold greater amounts of Cys than can GLAST or GLT-1 [49]. Based on these results, the physiological roles of EAAC1 in the central nervous system (CNS) would be involved in the neuroprotective roles mediated by GSH production [55].

## 5. Regulatory Mechanism of EAAC1 Expression in Neurons

While GLAST and GLT-1 are constitutively expressed on the cell membrane of glial cells, the membrane expression levels of EAAC1 are approximately 20% of the total under normal conditions, while protein kinase C (PKC) and phosphoinositide 3-kinase (PI3K) activations increase the EAAC1 expression on the plasma membrane [56] (Figure 3). On the other hand, Glu transporter-associated protein 3-18 (GTRAP3-18), which is an endoplasmic reticulum protein, binds to EAAC1 in the endoplasmic reticulum and suppresses the membrane trafficking of EAAC1 [57,58]. In our experiments both in vitro and in vivo, increased expression of GTRAP3-18 resulted in decreased GSH levels due to increased interaction with EAAC1 [59]. Subsequent experiments using antisense oligonucleotides and small interfering RNAs demonstrated that silencing the expression of GTRAP3-18 increased the GSH levels in neurons [59]. Indeed, in GTRAP3-18-deficient mice, the expression of EAAC1 on the cell membrane in neurons and both Cys and GSH levels in the brain tissues were also increased, leading to the resistance to oxidative stress [60]. These results suggest that suppression of GTRAP3-18 in neurons leads to resistance to neurodegeneration by promoting the function of EAAC1 to increase neuronal GSH synthesis. GTRAP3-18 hinders neurite outgrowth in vitro [61], while GTRAP3-18-deficient mice showed enhanced neurogenesis in the hippocampus [62] and spatial cognitive potentiation as assessed by the Morris water maze test [60,62]. Hippocampal neurons require GSH to sustain dendrite integrity and cognitive function [63]. Regulation of GTRAP3-18 would be a promising strategy to modulate neuronal GSH synthesis and thereby confer neuroprotection.

EAAC1 gene expression is promoted by nuclear factor erythroid 2-related factor 2 (Nrf2) [64], regulatory factor X1 (RFX1) [65], and all-trans-retinoic acid (ATRA) [66], while miR-96-5p, which is one of the microRNAs (miRNAs), has a target sequence in the 3’-UTR of EAAC1 and suppresses the protein expression of EAAC1 posttranslationally, leading to decreased GSH levels in the brain [67,68] (Figure 3). The function of EAAC1 is also promoted by the mammalian target of rapamycin (mTOR) [69] and Janus-activated tyrosine kinase-2 (JAK-2) [70], which are involved in cell growth, differentiation, and proliferation. On the other hand, activation of AMP-activated protein kinase (AMPK) reduces the expression of EAAC1 on the cell surface and suppresses its function [71]. AMPK is a serine-threonine kinase that is activated by cellular ATP depletion and is known to be involved in the maintenance of energy homeostasis by inhibiting anabolic action while promoting catabolism in cells. However, it is not clear how membrane translocation of EAAC1 is regulated by the activity of AMPK. Thus, it is quite probable that expression of EAAC1 is subject to pre- and post-translational regulations in neurons.

## 6. GSH Synthesis in Astrocytes

For the last 50 years, evidence has suggested that astrocytes outnumber neurons 10-fold and make up 25–50% of the brain volume [72,73], while recent papers have reported a glia:neuron ratio of less than 1:1 in the human brain [73]. In the brain, astrocytes play several important roles in maintaining physiological neuronal activity. Astrocyte-neuron interactions have been suggested to be crucial for neuronal survival [74,75]. Astrocytes promote the growth of neurites by releasing neurotrophic factors and reducing neurotoxicity by Glu uptake after brain injuries [76], while also protecting neurons from oxidative stress via a GSH-dependent mechanism [74,75]. Notably, GSH metabolic genes and GSH production in astrocytes can be up-regulated in neuronal co-culture through the modulation of astrocytic Nrf2 [77]. On the other hand, decreased GSH levels in astrocytes enhance neurotoxicity due to oxidative stress [75], indicating that neurons are more vulnerable to oxidative stress without a supply of Cys from astrocytes. 

The BBB prevents direct entry of GSH into the brain. GSH is oxidized to GSSG or decomposed to amino acids in blood, and the Cys in blood is easily oxidized to cystine. Astrocytes can take cystine into cells via a sodium-independent Glu/cystine antiporter named system xcˉ [78], which can exchange extracellular cystine for intracellular Glu and then intracellularly reduce cystine back to two Cys molecules that can be utilized as a substrate for GSH synthesis. Moreover, astrocytes can utilize the dipeptides γGluCys and CysGly for GSH synthesis, or convert Met to Cys via the transsulfuration pathway [79] to maintain high intracellular stores of GSH (approximately 8 mM) [29]. Astrocytes supply Cys-containing peptides to neurons in order to maintain GSH synthesis [80]. Astrocytes can release about 10% of their intracellular GSH per hour [81] to the extracellular space via multidrug resistance protein 1 (MRP1) [82]. Extracellular GSH is decomposed into CysGly by the astroglial ectoenzyme γ-glutamyl transpeptidase (GGT) [82]. Since CysGly is not directly taken up into neurons [81], CysGly is hydrolyzed by neuronal ectopeptidase into Cys and Gly [44,83], both of which are transported into neurons. Therefore, neuronal GSH synthesis depends on the system xcˉ and the GSH-supply mechanisms in astrocytes and is maintained by the mechanism of astrocyte-neuron interactions.

## 7. GSH Synthesis in Microglia

In the brain, microglia occupy about 5–12% of all cells and are more abundant in gray matter than white matter [84]. Microglia are activated in response to various injuries, such as ischemia, infection, inflammatory diseases, brain trauma, and neurodegenerative diseases. In contrast to astrocyte GSH synthesis, which plays a role in supporting neurons, microglial GSH synthesis appears to be exclusively focused on eliminating ROS generated under pathological conditions. GSH levels and their related enzyme activities, such as the activities of GPx and GR, are higher in cultured microglia than in cultured astrocytes and neurons, especially under oxidative stress conditions [85,86]. In addition, the microglia themselves, which are involved in the phagocytosis of dead cells and pathogens, produce O_2_·^−^ and NO when activated [87,88]. Therefore, microglia must have a sufficient defense mechanism against oxidative stress. GSH can suppress ONOOˉ production by directly reacting with O_2_·^−^ and NO, and can eliminate the cytotoxicity of H_2_O_2_ and peroxides by promoting the action of GPx. Microglia also express system xcˉ for GSH synthesis [89]. System xcˉ can take up extracellular cystine into the cell instead of excreting intracellular Glu out of the cell. The activated microglia express GLT-1 [90] and promote reuptake of the excreted Glu by system xcˉ for use in GSH synthesis [91]. An in vitro study demonstrated that the increase in microglial Glu uptake capacity was accompanied by an increase in intracellular GSH contents [89]. The results indicated that GLT-1 expressed in the vicinity of the system xcˉ was closely coupled to GSH production in microglia [89]. Since the microglia are exposed to large amounts of RON/RNS, especially under pathological conditions, the coupling between the system xcˉ and GLT-1 plays a critical role in microglial GSH synthesis against oxidative stress.

## 8. Brain GSH Levels in Neurodegenerative Diseases

Ageing is one of the risk factors involved in neurodegeneration, and both increased oxidative stress and decreased GSH levels are important risk factors for age-related neurodegeneration in the CNS [29]. Tissue GSH measurements in human autopsy brains have revealed that the total GSH pool is predominantly (>98.8%) in the reduced form, and the GSH levels of the gray matter (~0.83 mM) are lower than those of the white matter (~1.18 mM) [92]. Lower GSH levels in the gray matter, where neurons are rich, compared to those in the white matter could plausibly result in susceptibility to neurodegeneration due to oxidative stress. Moreover, hippocampal GSH levels in human postmortem brain samples have been reported to decrease with age [93], suggesting that they are implicated in the pathogenesis of Alzheimer’s disease (AD). However, GSH levels in postmortem brain samples are likely not fully representative of the original GSH levels in living patients, since they may change with time after death.

Proton magnetic resonance spectroscopy (^1^H-MRS) is a non-invasive technique for the detection of various neurochemicals, including GSH [94]. In normal adult volunteers, brain GSH levels are around 1–2 mM, with large variations depending on the region, gender, and age differences [95]. Brain GSH levels appear to be decreased in age-related neurodegenerative diseases such as AD, Parkinson’s disease (PD), and amyotrophic lateral sclerosis (ALS). Several studies have used ^1^H-MRS to investigate brain GSH measurements in patients with these neurodegenerative diseases.

For example, in the temporal and parietal lobes of healthy older adults, GSH levels measured by ^1^H-MRS were negatively correlated with amyloid β (Aβ) levels, as assessed by positron-emission tomography with the amyloid tracer Pittsburg compound-B (PiB) [96]. AD is the most common cause of dementia in the world. It is pathologically characterized by Aβ deposition and neurofibrillary tangles in the brain [97]. In the brains of patients with AD, increased oxidative stress due to abnormal aggregation of Aβ is considered to play a critical role in the onset of disease. Indeed, Aβ impairs EAAC1 function and suppresses Cys uptake [98]. Aberrant EAAC1 accumulation has been observed in degenerating neurons in AD brains, and is considered a specific feature of AD in the hippocampus [99]. In patients with AD or mild cognitive impairment, hippocampal GSH levels measured by ^1^H-MRS were significantly decreased compared to those of healthy older-age controls [100]. GSH levels were also found to be decreased in the frontal cortex of patients with AD, and the GSH reductions in these regions were correlated with the decline in cognitive functions [100]. 

PD is the second most common aging-related neurodegenerative disease after AD. PD is pathologically characterized by insolubilized α-synuclein accumulation in neurons and dopaminergic neurodegeneration in the substantia nigra of the midbrain. An initial study in the postmortem brains of PD patients reported decreased GSH levels in the substantia nigra of the midbrain [101], suggesting that the decrease in neuronal GSH levels may be a critical change prior to the onset of PD [102]. Exposure to certain neurotoxins has been suggested to be a risk factor for PD [103,104]. One of these neurotoxins, 1-methyl-4-phenyl-1,2,3,6-tetrahydropyridine (MPTP), is commonly used in an experimental PD model in vivo [105]. Our previous study using the MPTP mouse model of PD showed GSH depletions with increased oxidative stress and EAAC1 dysfunction in the midbrain [106]. These MPTP-induced neurotoxicities were prevented by pre-administration of n-acetylcysteine (NAC), a membrane-permeable Cys precursor [106]. A recent study using ^1^H-MRS demonstrated that intranasal administration of 200 mg of GSH significantly increased GSH levels in the dorsal putamen of patients with PD [107]. Many studies suggest that small polar molecules may be able to ‘bypass’ the BBB by nasal administration, indicating that the interface between the nasal cavity and the brain may be a more vulnerable part of the BBB [108]. Intranasal administration of reduced GSH could thus be an effective approach for delivery of GSH to the CNS.

ALS is also a neurodegenerative disease associated with oxidative stress [109]. The brains of ALS patients showed a 90% decrease of GLT-1 and a 20% decrease of EAAC1 compared to those of controls [110]. Recent clinical studies using ^1^H-MRS showed that GSH levels in the brains of ALS patients were decreased compared to those of age-matched healthy volunteers [111], and the decreased GSH levels in the motor cortex and corticospinal tract were inversely correlated with the time after diagnosis [112]. The decrease of GSH levels was more prominent in the motor cortex than in the white matter in ALS patients [112]. These results suggest that the brains of patients with ALS have limited antioxidant capacity.

Mutations in SOD1 cause ALS in humans [113], and the overexpression of the ALS-linked mutant hSOD1 also causes an ALS-like phenotype in rodents [114]. Hemizygous mice over-expressing wild-type hSOD1 (hSOD1WT) did not show the ALS-like phenotype, but did show it when crossed with GCLm-knockout mice, with a 70–80% decrease in total GSH levels [115]. These results indicate that GSH depletion enhances neurodegeneration in ALS models in vivo.

Transactive response DNA-binding protein 43 kDa (TDP-43) is an RNA-binding protein that abnormally accumulates in the motor neurons of ALS patients [116]. Mutations in the gene for TDP-43 cause familial ALS in humans and the ALS-like phenotype in transgenic animals [117]. Expression of the A315T mutant TDP-43 in vitro decreased GSH levels and increased both ROS and cell death, while the restoration of GSH levels by treatment with GSH monoethyl ester prevented cell death and TDP-43 pathological changes in motor neurons [118]. These results indicate that restoring GSH levels could be a promising strategy for the treatment of TDP-43-mediated ALS.

Multiple system atrophy (MSA) is an adult-onset neurodegenerative disease characterized by progressive cerebellar ataxia, autonomic symptoms, and parkinsonism. No radical treatment is available to prevent the onset or progression of MSA. Although the etiology has not been fully elucidated yet, the involvement of oxidative stress has been suggested as an important causative factor in recent years [119,120,121]. Recent papers in the field of neurodegenerative diseases have examined the posttranscriptional regulation of proteins by microRNA (miRNA) [68,122], and one of the miRNAs, named miR-96-5p, was particularly upregulated in the brains of MSA patients [123]. Our experimental results also showed that the increase in miR-96-5p causes a decrease in EAAC1 protein levels, leading to reduced GSH levels in neurons, while a treatment with anti-miR-96-5p restored the EAAC1 levels and increased GSH levels, leading to neuroprotective effects against oxidative stress in vitro and in vivo [67]. Moreover, anti-miR-96-5p indirectly decreased GTRAP3-18 protein levels [124]. For more details regarding the non-coding RNA-mediated regulatory mechanism of GSH synthesis, see our review article entitled “The role of non-coding RNAs in the neuroprotective effects of glutathione” by Kinoshita C. et al. in this special issue.

## 9. GSH Treatment for Neurodegenerative Diseases

The number of cases of age-related neurodegenerative diseases such as AD and PD are estimated to increase exponentially worldwide, and these diseases threaten to become a major clinical problem in the future. In recent years, many studies have been conducted with the goal of actively developing therapeutic agents for patients with these neurodegenerative diseases. In particular, there is a need for the development of “disease-modifying drugs” that suppress neurodegeneration, since none of the medicines clinically used at present provide radical therapeutic effects against the progression of these neurodegenerative diseases.

Since the 1990s, along with continued elucidation of the mechanism of neurodegeneration induced by oxidative stress in the CNS, GSH depletion in the brains of patients with neurodegenerative diseases has been increasingly reported. Subsequently, basic research on GSH in the CNS has been focused on therapeutic strategies aimed at reducing neurodegeneration, and a drug increasing GSH levels in the brain would be promising as a ‘disease-modifying drug’ characterized by neuroprotective effects. Since GSH hardly crosses the BBB [125], the clinical effects of direct GSH replacement therapy could not be expected to be neuroprotective. Orally administered GSH is not directly absorbed by the body because of its degradation by gastrointestinal peptidase. In addition, most of the intravenously administered GSH is also metabolized by GGT in the blood, so that the elimination half-life is as short as about 7 min [126], which is not sufficient for clinically effective administration. GSH in the blood is predominantly oxidized to GSSG under aerobic conditions, so that the administered GSH concentrations are lowered in the blood. It is difficult to increase the brain GSH levels directly by peripheral administration. Further studies on drug delivery technology will be needed in the future. 

In addition to GSH, some other antioxidants, such as ascorbic acid (vitamin C) and α-tocopherol (vitamin E), are also present in the brain. The concentrations of ascorbic acid in the brain are similar to those of GSH (about 1–2 mM) [127], but the reactivity of ascorbic acid to ONOOˉ is too low to provide neuroprotection [128]. The concentrations of α-tocopherol in the brain are lower than those of GSH or ascorbic acid, so that α-tocopherol is unlikely to play a central role among antioxidants [129]. Moreover, ascorbic acid and α-tocopherol, like GSH, hardly cross the BBB, so their brain concentrations cannot be increased by peripheral administration. In fact, the effectiveness of these antioxidants has not been clear in clinical studies of AD [130,131] and PD [132] patients. Indeed, no significant decreases in ascorbic acid or α-tocopherol levels were observed in the brains of AD and PD patients [129,133,134]. In addition, a clinical study applying coenzyme Q10, a mitochondrial antioxidant, did not demonstrate clinical efficacy in patients with early PD [135]. Although administration of some antioxidants may suppress neurodegeneration, no clinically apparent efficacy has been demonstrated yet. Among the antioxidants, GSH remains a promising agent because it is selectively decreased in the brains of patients with these neurodegenerative diseases. 

NAC is a membrane-permeable Cys precursor for GSH synthesis. NAC can diffuse into neurons without EAAC1 to supply Cys via intracellular deacetylation [52,136]. NAC also acts as an antioxidant [137] and stimulates GR, leading to a reduction of GSSG to GSH [138,139]. These results suggest a promising clinical application of NAC to increase neuronal GSH levels in the brains of patients with neurodegenerative diseases. A recent clinical trial with oral administration of NAC did not demonstrate increased GSH levels in some brain regions measured by ^1^H-MRS [140]. However, intravenous administration of NAC increased brain GSH levels by 55% in patients with PD [141]. These results indicate that an improvement of the drug-delivery system is necessary for treatment with NAC.

The BBB is a strict barrier in terms of protecting the CNS against toxic xenobiotics. Our paper published in 2021 [124] introduced a drug-delivery system that overcame the issue of the BBB by means of ultrasound combined with microbubbles containing anti-miR-96-5p; this system was recently shown to successfully realize neuroprotective effects by increasing brain GSH levels in vivo [124]. In our previous study, we found that the GTRAP3-18 levels were increased by miR-96-5p, which decreases EAAC1 levels in the brain [67]. We also found that intra-arterial injection of anti-miR-96-5p into mice using microbubbles and an ultrasound system decreased GTRAP3-18 levels, leading to increased EAAC1 and GSH levels in the hippocampus [124]. Recently, this drug-delivery system has received much attention as a new technology [142,143], and it might be useful for clinical application in the future. In combination with the development of drug-delivery systems, neuron-specific GSH replacement therapy holds promise for the future treatment of patients with neurodegenerative diseases.
